# Massive depletion of BLV proviral clones located in genomic transcriptionally active sites during primary infection

**DOI:** 10.1186/1742-4690-11-S1-O63

**Published:** 2014-01-07

**Authors:** Nicolas A Gillet, Gerónimo Gutiérrez, Alix de Brogniez, Sabrina M Rodriguez, Nathalie Renotte, Irene Alvarez, Lucas Vagnoni, Karina Trono, Luc Willems

**Affiliations:** 1 Molecular and Cellular Epigenetics, Interdisciplinary Cluster for Applied Genoproteomics (GIGA) of University of Liège (ULg), B34, Sart-Tilman Liège, Belgium; 2 Molecular and Cellular Biology, Gembloux Agro-Bio Tech, University of Liège (ULg), Gembloux, Belgium; 3 Instituto de Virología, Centro de Investigaciones en Ciencias Veterinarias y Agronómicas, INTA, C.C., Castelar, Argentina

## 

Only scarce data is available on early infection by human T-lymphotropic virus (HTLV). In particular, the modes of clonal selection during primary infection cannot be analyzed due to the paucity of available samples. Therefore, we addressed this question in a closely related animal model, bovine leukemia virus (BLV). As HTLV, BLV persist indefinitely into its host and is generally asymptomatic but can also lead to lymphoma or leukemia. Both viruses replicate by colonizing new lymphocytes or via clonal proliferation of infected cells. However, the modes of replication occurring soon after infection of new hosts are currently unknown. We used high-throughput sequencing to map and quantify the insertion sites of the provirus in order to monitor the clonality of the BLV-infected B-cell population (i.e. the number of distinct clones and abundance of each clone). We found that BLV propagation shifts from cell neoinfection to clonal proliferation in less than 3 months post-inoculation. Initially, BLV proviral integration significantly favors transcribed regions of the genome. Negative selection then eliminates more than 96% of the clones detected at seroconversion and disfavoring BLV-infected B-cells carrying a provirus located close to a promoter or a gene. This selection is more stringent in animals where proviral load set point is low. Among the surviving proviruses, clone abundance nevertheless positively correlates with proximity to a transcribed unit or a CpG island. We conclude that massive clone selection occurs during primary infection disfavoring proviruses located nearby genes and this selection is stronger in low proviral load animals.

